# Low Field Magnetic Stimulation Ameliorates Schizophrenia-Like Behavior and Up-Regulates Neuregulin-1 Expression in a Mouse Model of Cuprizone-Induced Demyelination

**DOI:** 10.3389/fpsyt.2018.00675

**Published:** 2018-12-06

**Authors:** Zuoli Sun, Tianhe Jiang, Yan Wu, Chao Ma, Yi He, Jian Yang

**Affiliations:** ^1^The National Clinical Research Center for Mental Disorders, Beijing Key Laboratory of Mental Disorders, Beijing Anding Hospital, Capital Medical University, Beijing, China; ^2^Key Laboratory for Neurodegenerative Disorders of the Ministry of Education, Capital Medical University, Beijing, China; ^3^Advanced Innovation Center for Human Brain Protection, Capital Medical University, Beijing, China

**Keywords:** schizophrenia, myelin sheaths, low field magnetic stimulation, neuromodulation, neuregulin-1

## Abstract

White matter and myelin sheath integrity are disrupted in schizophrenia, and non-invasive magnetic brain stimulation targeting these tracts is a promising new therapeutic approach. In particular, deep-brain reachable low field magnetic stimulation (DMS) could alleviate cognitive impairment and depressive-like behaviors in animal models. In this study, we sought to assess the effects of DMS on myelin sheath damage and schizophrenia-like behaviors in the cuprizone-induced demyelination mouse model. Mice were fed cuprizone (copper ion chelating agent, 0.2% w/w mixed with food) for 6 weeks to induce demyelination. During these 6 weeks, mice were stimulated with either sham, low-frequency (LFS, delta frequency) DMS or high-frequency (HFS, gamma Hz) DMS for 20 min each day. Behavioral tests were conducted 24 h after the final DMS session. The myelin sheath was examined by immunohistochemistry and the expression of neuregulin-1 (NRG1)/ErbB4 in the prefrontal cortex was measured with Western blotting. Six weeks of HFS significantly alleviated schizophrenia-like behaviors in cuprizone mice, including improved nesting, social interaction and sensorimotor gating, while LFS improved sensorimotor gating only. HFS and LFS both repaired the myelin sheath and increased the expression of neuregulin-1 and its receptor ErbB4, in the prefrontal cortex of demyelinated mice. Our findings show that DMS is a potential effective neuromodulation technique for the treatment of schizophrenia. One possible mechanism underlying these therapeutic effects could involve the up-regulation of NRG1/ErbB4 signaling in the prefrontal cortex.

## Introduction

Schizophrenia is a severe mental disorder with an estimated prevalence of 1% worldwide, and it is one of the leading causes of disability (1, 2). Schizophrenia is characterized by multidimensional clinical features, including positive symptoms, negative symptoms, and cognitive impairment (3). Antipsychotic medications are the mainstream treatment for schizophrenia, but many patients do not respond to treatment or have poor outcomes despite symptom improvement with antipsychotic medication (4).

Repetitive transcranial magnetic stimulation (rTMS) is a safe, non-invasive, and well-tolerated clinical technique to modulate brain function. Because there are few side effects, rTMS is becoming increasingly important in the treatment of psychiatric disorders (5). For schizophrenia, clinical evidence shows rTMS has therapeutic effects on persistent auditory hallucinations and negative symptoms. However, the efficacy of rTMS has often been shown to be modest, perhaps because stimulation is limited to superficial brain areas, mainly the cortex (6).

In contrast, deep-brain reachable low field magnetic stimulation (DMS) produces a magnetic field with high output frequency, low intensity, and wide range that can stimulate deeper brain regions such as the hippocampus (7). DMS stimulates the brain by emitting electromagnetic fields, similar to those produced by echo planar magnetic resonance imaging (8). Low field magnetic stimulation produced rapid improvements in mood in depressed patients in a randomized controlled study (9), which could be mediated by increasing brain-derived neurotrophic factor (BDNF) levels (10). DMS has also been shown to improve cognitive function in a transgenic mouse model of Alzheimer's disease (7). In this study, we sought to investigate the possible impact of DMS on schizophrenia-related features in a mouse model that simulates white matter and connectivity deficits of schizophrenia.

rTMS acts through many mechanistic pathways, including regulation on neuronal function, neurotransmitter release, neurotrophic effects, synaptic plasticity, oxidative stress, and inflammation (11). However, there have been limited research on how rTMS might modulate oligodendrocytes and the myelin sheath. Schizophrenia has been associated with mutations in oligodendrocyte-related genes and diffusion tensor imaging (DTI) shows abnormal white matter tracts (12–17).

Myelination of axons by oligodendrocytes is essential for rapid and accurate conduction of electrical impulses in the central nervous system, which is very important for proper physiological function (18). Disruption of myelin development may interfere with communication between neurons (19). Myelination is under complex regulation, involving growth factors as well as their downstream signals such as neuregulin (NRG) (20, 21). Both electric and magnetic fields can enhance oligodendrocyte myelination capacity and improve the differentiation of oligodendrocyte precursor cells into new oligodendrocytes, and this may be mediated by enhanced neurotrophic factor release (22–24).

Although clinical studies have demonstrated the efficacy of magnetic stimulation on schizophrenia symptoms, relatively few studies have investigated the effects of magnetic stimulation on white matter. Based on the data reviewed above, we hypothesized that DMS could improve schizophrenia-like behaviors in mice through acting on myelinated white matter tracts, and the molecular mechanism might involve the regulation of NRG1 signaling. Our study was set to examinethe effects of DMS treatment on schizophrenia-like behaviors, as well as the expression of NRG1 in brain areas relevant to schizophrenia.

## Materials and Methods

### Animals and Equipment

Six-week-old male C57BL/6 mice were supplied by the laboratory animal center at Capital Medical University, Beijing, China. These mice were placed in a standard 12 h light-dark cycle with free access to food and water. The protocol was approved by the Animal Use and Care Committee at Capital Medical University. After 7 days of acclimatization, the mice were randomly and evenly distributed to the following four groups (*n* = 12 per group): control, model, model + low frequency DMS (LFS) and model + high frequency DMS (HFS). The detailed frequency paradigm was described below. The control mice received normal rodent chow, while the model mice were fed with rodent chow containing 0.2% (w/w) cuprizone (CPZ, Sigma Aldrich, St. Louis, MO, USA) for 6 weeks to induce demyelination (25). During these 6 weeks, the mice in therapeutic groups were subjected to DMS treatment at the same time. The behavioral tests were conducted the following day after the final intervention, and then the animals were killed immediately. About half of the mice were sacrificed and the expression levels of NRG1 and ErbB4 in the prefrontal cortex were determined by Western blot. The other mice were perfused and the myelin sheath was visualized by immunohistochemistry.

**Figure 1:**
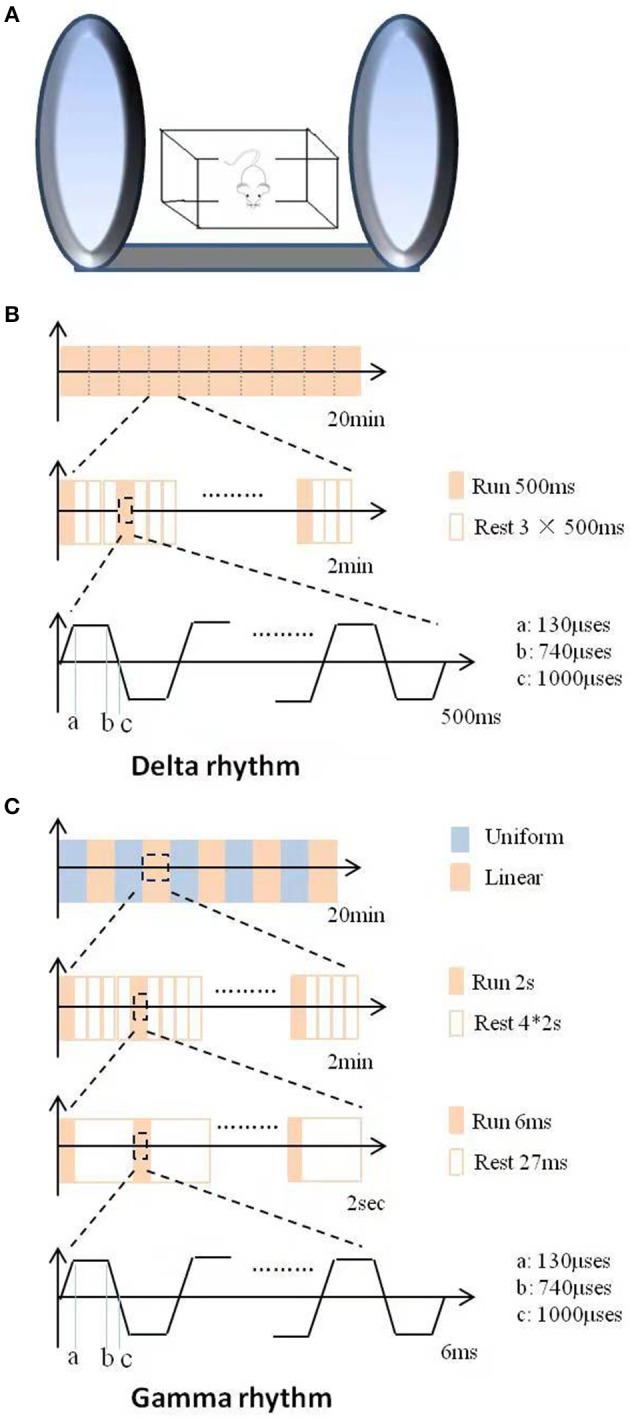
Schematic illustration of the DMS apparatus and stimulus patterns used in this study. **(A)** The apparatus and operational principle of DMS. **(B)** The stimulus pattern of LFS with delta rhythm. **(C)** The stimulus pattern of HFS with gamma rhythm.

For DMS treatment, the mice were placed in the magnetic device (provided by Beijing Aldans Biotech Co, Ltd, Figure 1A), consisting of two 360 mm-diameter coils connected to a magnetic field generator. Twenty minutes of successive trains of DMS were administered daily for 6 continuous weeks. Intermittent gamma (Figure 1C) or delta (Figure 1B) burst stimulation was used. The magnetic flux density was a uniform alternating linear gradient in the gamma paradigm (high frequency), which was divided into ten 2-min outputs composed of 2-s outputs and 8-s resting intervals. Each 2-s output was consisted of cyclic 6-ms train of spikes and intervals. Each train spike was composed of 6 successive pulses, and the intervals were 27, 25, 23, 21, or 19-ms, which changed every 4 min. Contrast to the gamma paradigm, the magnetic flux density was a linear gradient in delta paradigm (low frequency). The 2-min output was consisted of a 500 ms running time followed by a 1,500 ms resting time and repeats.

### Behavioral Paradigms

Behavioral tests were conducted 24 h after the final DMS treatment.

#### Open-Field Activity

The Open-field test is an established tool to determine spontaneous activity (measured as the distance and speed of exploratory behavior in a novel environment) and anxiety-like behaviors (measured as the time spent in the open central area). Spontaneous locomotor activity was measured using a movement detection system (Shanghai Xinruan Information Technology, Co. Ltd, China). Each mouse was placed in a cage alone (40 × 40 × 25 cm) for 13 min: 3 min was for adaptation and 10 min for recording activity. The total distance (cm) and central area distance traveled (cm) during the 10-min assessment period was recorded and analyzed using a video tracking system. The cage was wiped with ethanol to remove odor between trials.

#### Nest Construction

The nest construction assay was used to assess affiliative social behavior. A compressed cotton square (5 × 5 cm) was placed into each home cage at 6 pm. The next morning, cages were inspected and photographed to document nest construction according to a 1–5 point rating scale as follows (26): (1) nest cotton was untouched (> 90% intact); (2) nest cotton was scattered (50–90% remaining intact); (3) nest was built roughly (50–90% of nesting material torn); (4) nest was probably formed (>90% of the cotton is torn, but the nest is flat); (5) a near perfect nest (>90% of the cotton is torn, and the nest is a crater with enclosing walls).

#### Social Interaction Test

The social interaction test was used to assess social approach behavior and interest in social novelty (27). Each apparatus (40 × 60 × 20 cm) was divided into three small compartments. Before testing, each mouse was habituated to the apparatus for 5 min, once per day for 3 consecutive days. On the testing day, in trial 1, the subject mouse was placed in the middle chamber and allowed to explore all three chambers for 10 min. An unfamiliar mouse enclosed in a wire cage was placed in one side chamber and an empty cage was placed in the other side chamber. In trial 2, a new unfamiliar mouse was placed in the previously empty side chamber. The movement and time spent interacting with the mice in the side chambers was recorded and analyzed with EthoVision (EthoVision XT 9.0, Netherlands).

#### Pre-Pulse Inhibition of Acoustic Startle (PPI)

Auditory startle reflex and sensory gating was evaluated using commercial startle chambers (MED-ASR-PRO1, MED Associates Inc, USA). During testing, each animal was held in a clear transparent cylinder connected to a piezoelectric sensor. A continuous 60 dB background white noise was maintained throughout testing, via a ceiling speaker located within the chamber. After 5 min acclimatization with 60 dB background noise, the PPI test session with a total of 80 trials (20 s intertrial interval) was conducted. Each of the first 20 trials consisted of a single 20 ms 110 dB startle pulse alone stimulus. The remaining 60 trials consisted of six types of prepulse + pulse trials as follows: 2 ms duration prepulse at 75 or 85 dB, followed by no startle stimulus or a 20 ms duration startle stimulus at 110 dB with a 30 or 100 ms delay. Each trial type was repeated 10 times in a random manner. The percentage of PPI was calculated according to the following formula: PPI = (1 − prepulse plus startle amplitude/startle only amplitude) × 100. The PPI sessions were carried out between 08:00 and 12:00 am.

### Tissue Preparation and Immunohistochemical Staining

After behavioral tests, six mice in each group were anesthetized with sodium pentobarbital (50 mg/kg) and perfused intracardially with 0.9% saline followed by 4% paraformaldehyde (PFA) in saline. Serial coronal sections (30 μm) of the brain were made on a freezing microtome and then processed for immunohistochemistry using an anti-myelin basic protein (MBP) rabbit polyclonal antibody (1:200 dilution, Sigma Aldrich, St. Louis, MO, USA). Images were obtained using a Nikon BX-51 light microscope.

### Western Blot Analysis

After behavioral tests, six mice in each group were sacrificed by cervical dislocation, their brains were rapidly removed and frozen at −80°C. The prefrontal cortex was dissected and homogenized with a tissue grinding instrument (DHS Life Science, Beijing, China). The tissue was lysed with Tris–EDTA lysis buffer (20 mM Tris, pH 7.5, 1% Triton X-100, 10% glycerol, 1 mM EDTA) containing protein cocktail inhibitors (Sigma Aldrich, St. Louis, MO, USA). Proteins were separated with sodium dodecyl sulfate polyacrylamide gel electrophoresis and transferred to Immobilon-FL NC membranes (Millipore UK Ltd, Watford, UK). Beta-actin (1:1,000; Santa Cruz, USA) was used as an internal loading control, and anti-MBP (1:1,000; Sigma Aldrich, St. Louis, MO, USA) was used to detect the expression of MBP. After incubation with a secondary antibody (peroxidase-coupled antirabbit antibody, 1:1,000), the reactive signals were revealed by enhanced chemiluminescence (ECL system, Bio-Rad, USA). Images were captured using v3 workflow (Bio-Rad, USA) and band densitometric analysis was performed using Image Lab Software (Bio-Rad, USA).

### Statistical Analysis

Data analysis was performed by the GraphPad software (Prism 6 version). The results were expressed as means ± SEM. Group differences were determined using the one-way analysis of variance (ANOVA), followed by the Student–Newman–Keuls *post-hoc* test for multiple comparisons. For the social interaction behavior analysis, a two-way analysis of variance was applied. A probability of *P* < 0.05 was considered to be statistically significant.

## Results

### Schizophrenia-Like Behaviors

**Figure 2:**
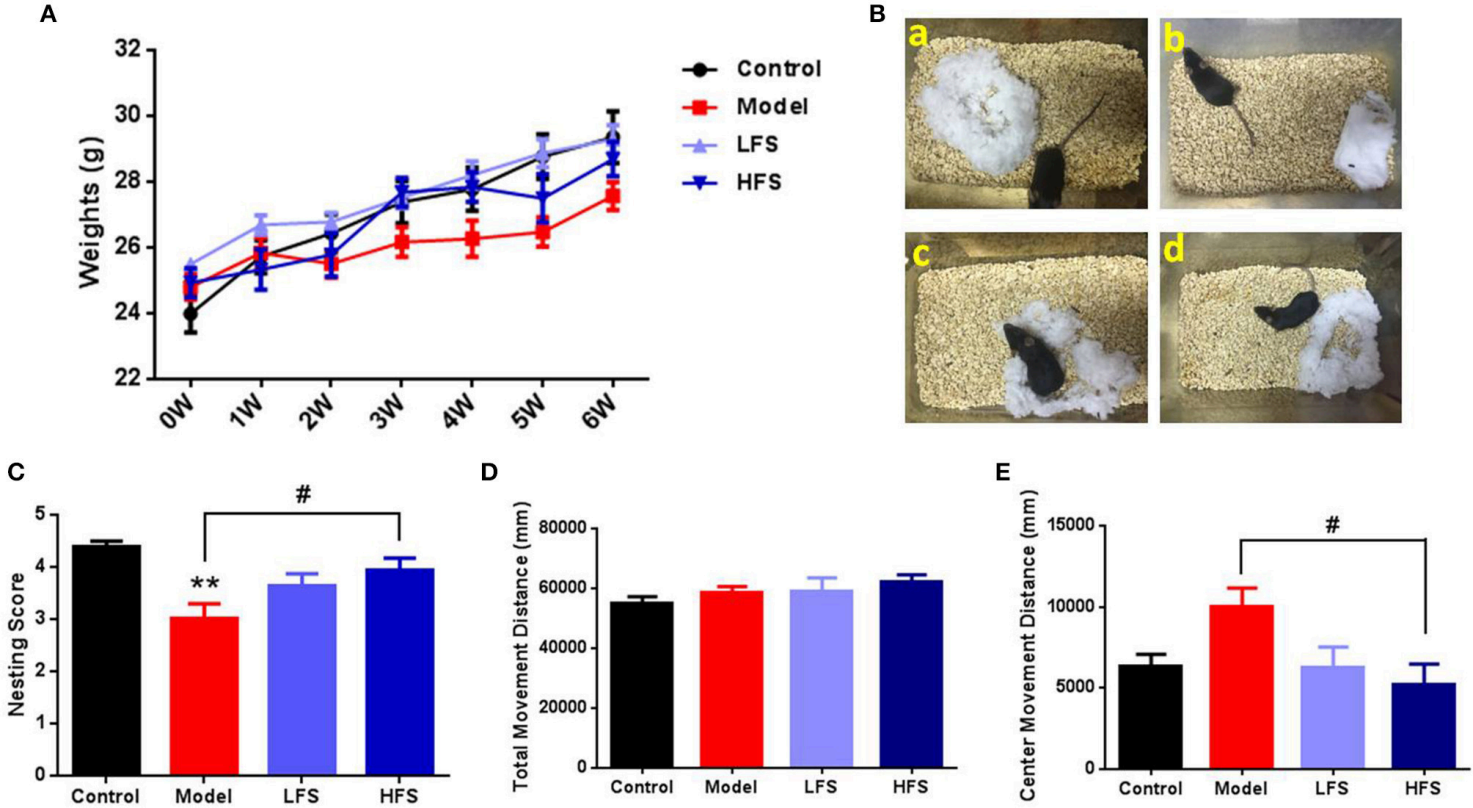
Body weight, nesting, and locomotion are altered by cuprizone and partially rescued by DMS. **(A)** Weight changes in mice subjected to different treatments. **(B)** Representative images of nests built by control **(a)**, model **(b)**, LFS **(c)**, and HFS **(d)** mice. **(C)** Histogram of nesting scores. **(D)** Total movement distance in 10 min. **(E)** Center zone movement distance in 10 min. Values are expressed as mean ± SEM (*n* = 12–15). ^**^P < 0.01 vs. control group; ^#^*P* < 0.05 vs. model group.

Cuprizone administration induced sustained weight loss compared with the control group, which was reversed by both LFS and HFS treatment (Figure 2A). Six weeks of cuprizone administration induced behaviors in mice that are related to schizophrenia, including abnormal nesting, locomotion, social interaction, and PPIs. Compared with control mice, cuprizone-treated mice had significant defects in nest-building (*P* < 0.01). HFS significantly increased nesting scores (*P* < 0.05) to the same level as control mice, whereas LFS had no significant effect on nesting (*P* > 0.05) (Figures 2B,C). In the open-field test, there were no significant differences in the total movement distance among the four groups, indicating similar locomotor activity (Figure 2D). However, when only considering the movement distance in the center zone, which is evaluated as a measure of anxiety, cuprizone-treated mice revealed a remarkable trend of increase compared with control mice (*P* > 0.05), indicating a diminished anxiety trait. Both HFS and LFS could reverse this change, but only HFS showed statistical difference (*P* < 0.05) (Figure 2E).

**Figure 3:**
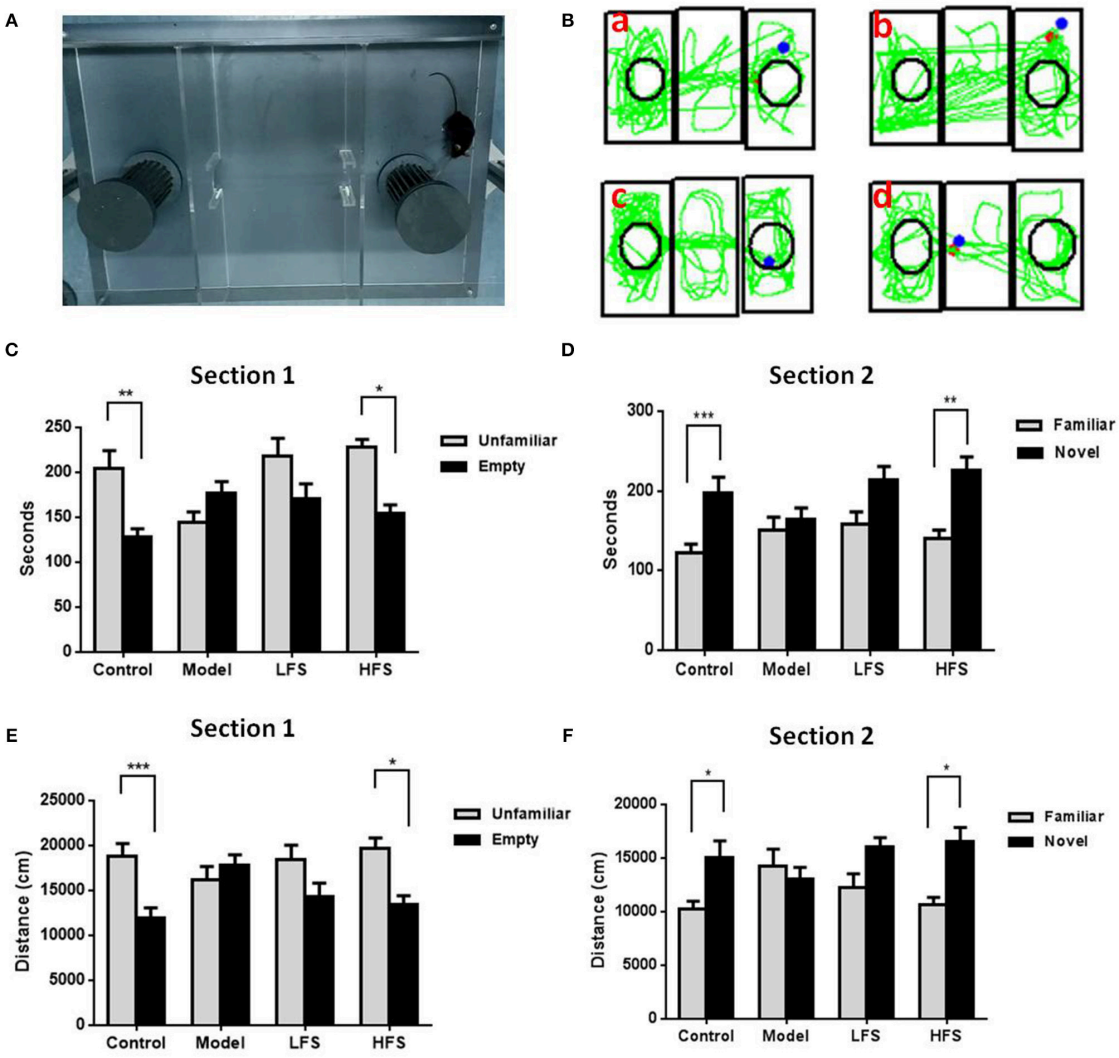
Social behavior is impaired by cuprizone and rescued by DMS. Results of the social interaction test are shown in **(A–D)**. **(A)** Photo of the three-chamber apparatus used for the social interaction test. **(B)** Representative movement traces of **(a)** control mice, **(b)** model group, **(c)** LFS group, and **(d)** HFS group. **(C,D)** Histograms showing time spent in each side chamber in trials 1 and 2. **(E,F)** Histograms showing the distance traveled in proximity to the wire cages containing stranger mice or the empty cage in trial 1. Values are expressed as mean ± SEM (*n* = 12–15). ^*^P < 0.05, ^**^P < 0.01, ^***^P < 0.001.

Social interaction tests are used to assess social withdrawal behavior, which is a core negative symptom of schizophrenia. In trial 1, control mice spent more time interacting with the stranger mouse than with the empty cage (Figure 3, *P* < 0.01). In trial 2, control mice preferred to be near the new stranger mouse than the familiar one (*P* < 0.05). In contrast, cuprizone-treated mice showed no preference between the empty cage and the stranger mouse in both trials 1 and 2. HFS, but not LFS, increased interaction time with stranger mice in both trials (both *P* < 0.05).

**Figure 4:**
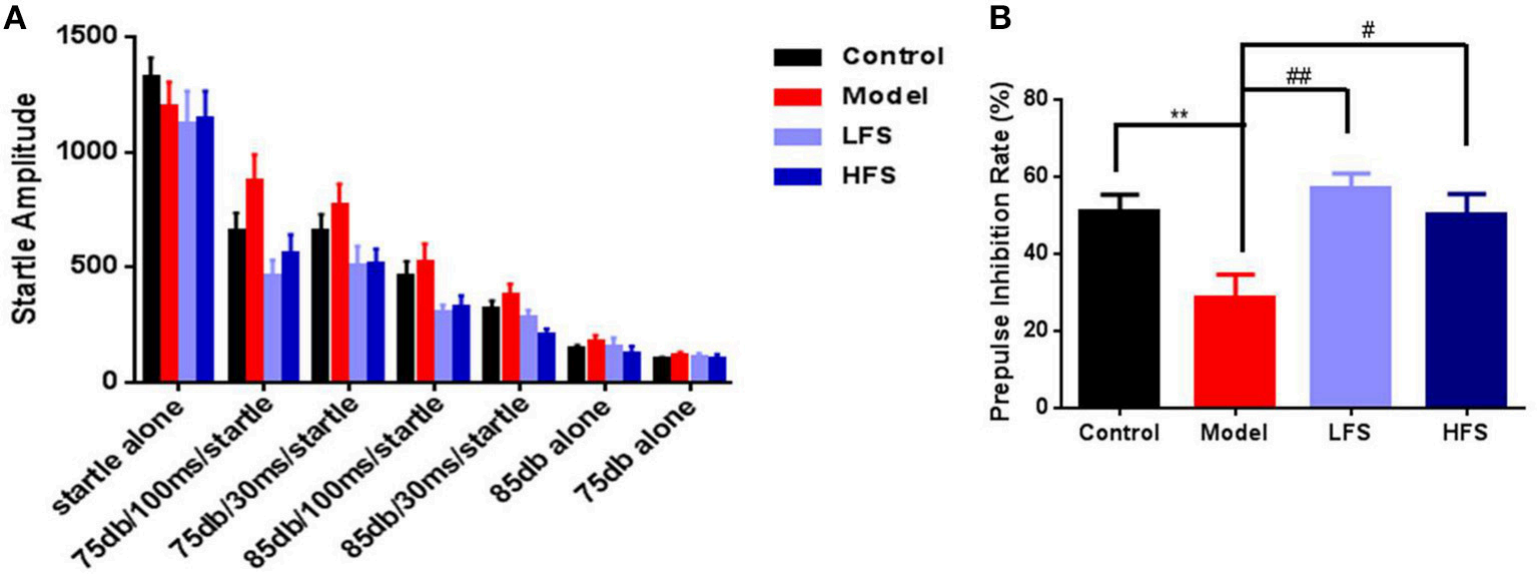
PPI deficits in cuprizone mice are rescued by DMS. **(A)** Startle amplitude for all PPI test epochs. **(B)** PPI at 75 dB with 100 ms interval. Values are expressed as mean ± SEM (*n* = 12–15). ^**^P < 0.01 vs. control group; ^#^*P* < 0.05, ^##^*P* < 0.01 vs. model group.

Impaired sensory gating is a heritable and well-studied endophenotype for schizophrenia (28, 29). In this study, six types of pre-pulse modes were used. The startle amplitude decreased with the increase of pre-pulse stimulation (Figure 4A). The cuprizone-treated mice showed a trend toward decreased PPI, especially with a pre-pulse intensity of 75 dB and a 100 ms interval (Figure 4B, *P* < 0.01). With these parameters, both LFS (*P* < 0.01) and HFS significantly increased PPI (*P* < 0.05).

### Myelin Sheath Integrity

**Figure 5:**
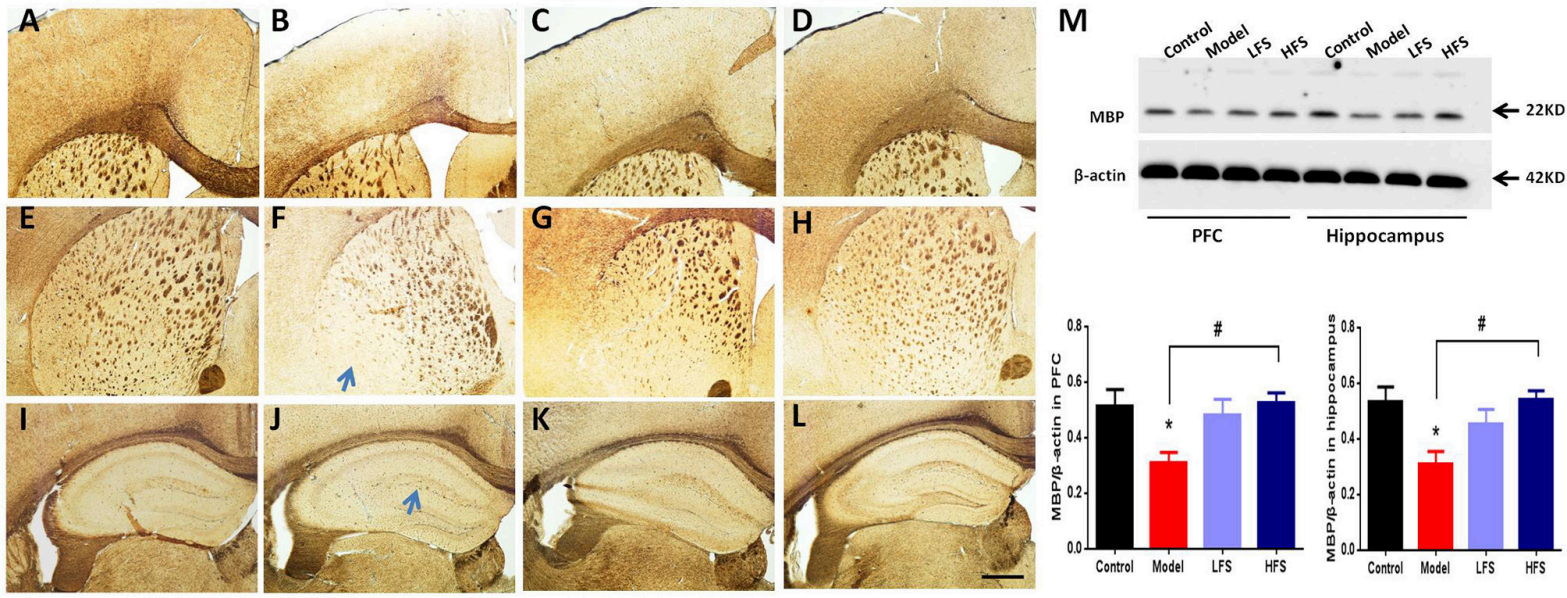
DMS restores myelination defects due to cuprizone. Effects of 6 weeks of DMS treatment on MBP protein expression are shown. MBP staining in cortex is shown in **(A–D)**. **(A)** Control group. **(B)** model group. **(C)** LFS group. **(D)** HFS group. MBP staining in the striatum is shown in **(E–H)**. **(E)** Control group. **(F)** Model group. The blue arrow points to the lateral striatum. **(G)** LFS group. **(H)** HFS group. MBP staining in the hippocampus is shown in **(I–L)**. **(I)** Control group. **(J)** Model group. The blue arrow points to the dentate gyrus. **(K)** LFS group. **(L)** HFS group. Scale bar = 100 μm. **(M)** Western blot and quantitative analysis of MBP expression in the prefrontal cortex and hippocampus. β-actin was used as an internal control. Values are expressed as mean ± SEM (*n* = 4–5). ^*^P < 0.05 vs. control group; ^#^*P* < 0.05 vs. model group.

To determine whether of the behavioral effects of DMS in cuprizone-treated mice is associated with anti-demyelination effects, we examined the expression of MBP in several brain regions. As shown in Figures 5A–L, MBP staining revealed significant myelin sheath loss in the cortex, corpus callosum, striatum, and hippocampus (especially in dentate gyrus) in cuprizone-treated mice. In particular, these demyelinated mice had significantly decreased radial distribution of MBP in the cortex, thinner corpus callosum and remarkable loss of fiber bundles in the lateral striatum. Both HFS and LFS could rescue these myelination deficits, in which HFS seemed to have stronger anti-demyelination effect than LFS. Immunoblotting results also demonstrated that both HFS and LFS could increase the expression of MBP in the prefrontal cortex and hippocampus in cuprizone-treated mice to a similar extent (Figure 5M). These results indicate that DMS can protect against demyelination induced by cuprizone.

### Expression of NRG1 and ErbB4 in Prefrontal Cortex

**Figure 6:**
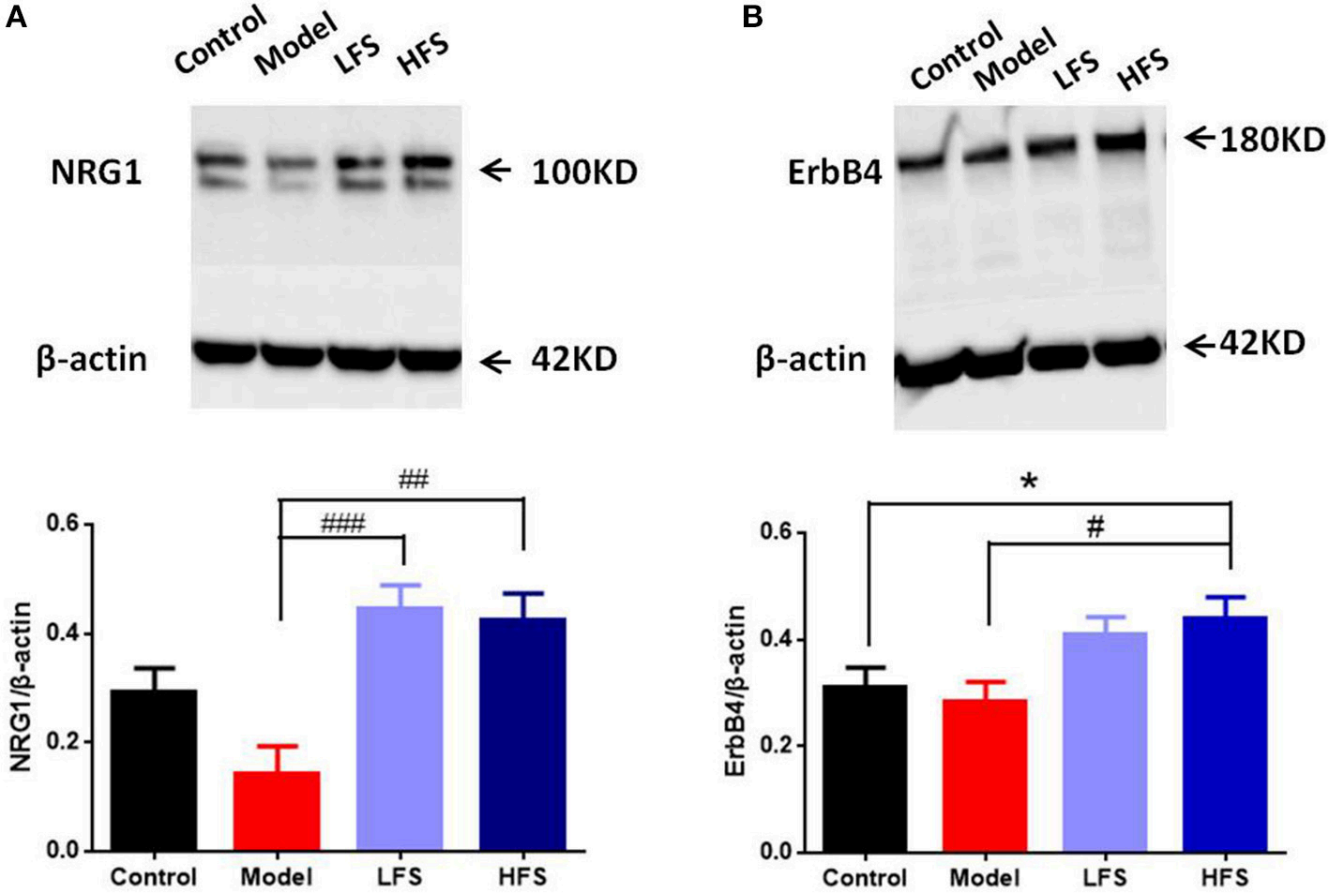
DMS treatment restores deficient NRG1 and ErbB4 expression in cuprizone mice. **(A,B)** Western blot and quantitative analysis of **(A)** NRG1 and **(B)** ErbB4 protein levels in the prefrontal cortex of mice. The upper figure shows the Western blot. β-actin was used as an internal control. The bottom panel is a histogram of the quantitative values from the corresponding blot. Values are expressed as mean ± SEM (*n* = 4). ^*^P < 0.05 vs. control group; ^#^*P* < 0.05, ^##^*P* < 0.01, ^###^*P* < 0.001 vs. model group.

NRG1-ErbB4 signaling is crucial for regulating oligodendrocyte development and CNS myelination (30). Since prefrontal cortex is one of the most schizophrenia-related regions, we took it as an example to examine the possible effects of DMS on NRG1-ErbB4 signaling in the brain. Results of immunoblotting showed that cuprizone significantly decreased NRG1 levels in the prefrontal cortex, with a lesser effect on ErbB4. Both HFS and LFS restored the expression of NRG1 and ErbB4 in the prefrontal cortex in cuprizone-treated mice, with no significant differences between the two types of DMS (Figures 6A,B). These results suggest that the protective effects of DMS on demyelination could be caused by enhancing NRG1-ErbB4 signaling.

## Discussion

We investigated the effects of two different DMS paradigms on behavior, histology and protein expression in experimentally demyelinated mice as a model for some aspects of schizophrenia. Both LFS and HFS improved schizophrenia-like behaviors in cuprizone-treated mice, but with disparate effects. LFS significantly restored sensory gating impairments, while HFS normalized nesting ability, locomotor activity, social interaction, and sensory gating. In addition, DMS rescued demyelination, and increased the expression of MBP in the cortex, corpus callosum, striatum, and hippocampus. Furthermore, DMS restored the decrease in both NRG1 and ErbB4 in cuprizone-treated mice.

Our data confirmed that demyelination can cause a mouse behavioral phenotype related to schizophrenia and our findings are consistent with clinical observations of patients with schizophrenia, including the disrupted connectivity and white matter integrity. The myelin sheath is essential to provide nutritional support for the axon and increase the conduction velocity of action potentials, which is very important for physiological functioning (18). Disruption of myelin development may interfere with communication between neurons (19). Therefore, the evolutionary increase of white matter content in the human brain may have contributed to our superior cognitive abilities compared to other animals. A recent study indicated that mice with a subtle defect of myelination could exhibit the schizophrenia-like symptoms (31). In the past decade, there is growing evidence from imaging (32), genetic (33, 34), and postmortem (35, 36) studies that white matter impairment plays an important role in the pathogenesis of schizophrenia.

Cuprizone, a copper ion chelating agent, is able to induce oligodendrocyte loss and demyelination in the CNS of mice (37). The degree of demyelination induced by cuprizone was different in several schizophrenia-associated brain regions. For example, the myelin sheath displays obvious ruptures in cerebral cortex, hippocampus and olfactory bulb, but the effects of cuprizone is relatively mild in thalamus and hypothalamus (38). Recent studies reported that low dose cuprizone induces behavioral deficits similar to symptoms in human schizophrenia, including reduced anxiety-like responses to novelty (37), deficits in learning and memory (37, 39), lower pre-pulse inhibition (40), and decrease in social activity (41). Therefore, cuprizone-treated mice have been widely used as a novel animal model of schizophrenia to explore the mechanisms of schizophrenia-associated white matter abnormalities.

Consistent with this line of reasoning, our results confirmed that chronic cuprizone treatment in mice not only can produce remarkable loss of myelin in the frontal cortex, corpus callosum, and striatum, it can also induce schizophrenia-like behavioral abnormalities, such as deficits in nest construction, social interaction, and PPI. Moreover, we observed a slight hyperactivity of cuprizone-treated mice in the open field test. This could be interpreted as a potential link between dopamine dysfunctions and myelin deficits (42). Up to now, there is still no mouse model that can capture all the features of a complex disorder like schizophrenia (43, 44), our data indicate that cuprizone can induce schizophrenia-like behaviors with related pathology in white matter.

Several clinical studies have shown the therapeutic effects of magnetic stimulation on schizophrenia, but the specific effects of each paradigm vary. Many studies investigated high-frequency rTMS as a way to increase activation of the left prefrontal cortex for the treatment of negative symptoms (45–47). In addition, temporoparietal low-frequency rTMS is commonly used to treat auditory hallucinations (48). A randomized, double-blind, sham-controlled study showed that cerebellar high frequency rTMS could be an effective adjunct therapy for negative and affective symptoms (49). Recently, a meta-analysis reported 1-Hz rTMS was effective in reducing auditory hallucinations in schizophrenia, while 10-Hz rTMS did not differ significantly from sham in the improvement of negative symptoms (50). In this study, we found that both LFS and HFS were capable of rescuing schizophrenia-like behaviors, but HFS seemed to be more potent than LFS. LFS only significantly relieved sensory gating deficits, while HFS showed broader effects in improving nesting ability, social interaction and sensory gating. As schizophrenia is associated with cortical hyperexcitability and inhibition deficit, the different behavioral effects of HFS and LFS might relate to their different role in regulating cortical activity and network connections. It has been suggested that rTMS could enhance cortical inhibition, in which HFS revealed a significant lengthening compared to LFS (51). In addition, a recent study evaluated the intranetwork and internetwork effects of rTMS with LFS or HFS using functional magnetic resonance imaging (fMRI). They found HFS could lead to broader effects on brain functional network compared with LFS (52). These results might explain the better effects of HFS on behavioral tests in our study.

NRG1, a neurodevelopmental regulator that can be neuroprotective against perinatal brain white matter injury (53). NRG1-ErbB4 signaling is involved in oligodendrocyte development, including cell migration, differentiation, maturation, and myelination. In addition, NRG1 is also required for adult nerve repair and remyelination (30). The downstream pathways of NRG1-ErbB4 signaling may be related to PI3K-Akt (54). In the present study, we found a downregulation of NRG1-ErbB4 signaling in cuprizone-treated mice. This result is consistent with the effects of MK-801, which is a very common pharmacological animal model of schizophrenia. MK-801 can induce obvious schizophrenia-like behaviors while also decreasing NRG1 in the mouse (55). Reduced NRG1-ErbB4 signaling was also found in patients with first-episode schizophrenia (56). However, there are some contradictory reports. A post-mortem study of schizophrenia found elevated NRG1 and ErbB4 proteins in the prefrontal cortex (57). Feng et al. reported increased NRG1β and ErbB4 expression in rat prefrontal cortex and hippocampus after injection with MK-801 (58). These contradictory results could be explained by differences in the actual NRG1 and ErbB4 levels between early and late phases of schizophrenia, the severity of disease and the tissues sampled. For example, in the animal studies, differences in rat vs. mouse, and the duration and dosage of MK-801 could have affected the results. Notably, we found that the expression of NRG1 was obviously, despite not statistically significantly decreased in the cuprizone-treated mice compared to the controls, but ErbB4 levels were almost unaltered. This inconsistent changes of NRG1 and ErbB4 were similar to that reported by Li et al. using the MK-801 model (55), which might be partly due to compensatory increase of ErbB4 after NRG1 reduction.

Although the efficacy of magnetic stimulation on schizophrenia has been confirmed by many studies, few have focused on the effects on white matter (11, 22). A recent study showed that the specific stimulation site had different effects on brain network activity. For instance, bilateral rTMS exerted a significant inhibitory effects (23). Our study found myelin sheath loss in several brain areas, including cerebral cortex, corpus callosum, and striatum, was alleviated by magnetic stimulation in cuprizone-treated mice, confirming the protective effects of magnetic stimulation on white matter. This is consistent with Sherafat et al. who reported that electromagnetic fields (60 Hz; 0.7 mT, 2–4 weeks) significantly reduced demyelination and increased MBP staining in an animal model with demyelination (24). One recent *in vitro* study showed that 2 weeks of static magnetic field enhanced myelination capacity and increased the secretion of neurotrophic factors (BDNF and NT-3) in a dorsal root ganglion microfluidic chamber platform (22). DMS both in alpha and delta rhythm improved depressive symptoms in patients while also increasing serum levels of BDNF (10).

In summary, we found that both LFS and HFS over 6 weeks significantly improved schizophrenia-like behaviors in cuprizone-treated mice. Specifically, LFS corrected deficits in sensory gating, while HFS significantly improved the nesting, social interaction and sensory gating. DMS also restored myelination and MBP expression in the cerebral cortex, corpus callosum, and striatum of cuprizone-treated mice. These behavior and cellular changes were accompanied by rectification of decreased NRG1 and ErbB4 expression in the prefrontal cortex. These results suggest that DMS may repair white matter deficits through neurotrophic mechanisms. Further experiments need to be conducted in the future to explore the molecular mechanisms underlying the therapeutic effects of DMS on schizophrenia.

## Author Contributions

JY and ZS designed the research and obtained funding for this study. TJ, YW, CM, and YH performed research. TJ and ZS analyzed data. ZS and JY wrote the paper.

### Conflict of Interest Statement

The authors declare that the research was conducted in the absence of any commercial or financial relationships that could be construed as a potential conflict of interest. The reviewer YZ declared a shared affiliation, with no collaboration, with several of the authors, ZS, TJ, YW, CM, YH, JY, to the handling editor at the time of the review.
